# Functional Adaptation of a Plant Receptor- Kinase Paved the Way for the Evolution of Intracellular Root Symbioses with Bacteria

**DOI:** 10.1371/journal.pbio.0060068

**Published:** 2008-03-04

**Authors:** Katharina Markmann, Gábor Giczey, Martin Parniske

**Affiliations:** 1 Genetics, Faculty of Biology, Ludwig Maximilians Universität, Munich, Germany; 2 The Sainsbury Laboratory, John Innes Centre, Norwich, United Kingdom; Max Planck Insitute for Developmental Biology, Germany

## Abstract

Nitrogen-fixing root nodule symbioses (RNS) occur in two major forms—Actinorhiza and legume-rhizobium symbiosis—which differ in bacterial partner, intracellular infection pattern, and morphogenesis. The phylogenetic restriction of nodulation to eurosid angiosperms indicates a common and recent evolutionary invention, but the molecular steps involved are still obscure. In legumes, at least seven genes—including the symbiosis receptor-kinase gene *SYMRK*—are essential for the interaction with rhizobia bacteria and for the Arbuscular Mycorrhiza (AM) symbiosis with phosphate-acquiring fungi, which is widespread in occurrence and believed to date back to the earliest land plants. We show that *SYMRK* is also required for Actinorhiza symbiosis of the cucurbit *Datisca glomerata* with actinobacteria of the genus *Frankia*, revealing a common genetic basis for both forms of RNS. We found that *SYMRK* exists in at least three different structural versions, of which the shorter forms from rice and tomato are sufficient for AM, but not for functional endosymbiosis with bacteria in the legume Lotus japonicus. Our data support the idea that *SYMRK* sequence evolution was involved in the recruitment of a pre-existing signalling network from AM, paving the way for the evolution of intracellular root symbioses with nitrogen-fixing bacteria.

## Author Summary

As an adaptation to nutrient limitations in terrestrial ecosystems, most plants form Arbuscular Mycorrhiza (AM), which is a symbiotic relationship between phosphate-delivering fungi and plant roots that dates back to the earliest land plants. More recently, a small group including the legumes and close relatives has evolved the ability to accommodate nitrogen-fixing bacteria intracellularly. The resulting symbiosis is manifested by the formation of specialized root organs, the nodules, and comes in two forms: the interaction of legumes with rhizobia, and the more widespread Actinorhiza symbiosis of mostly woody plants with *Frankia* bacteria. The symbiosis receptor kinase SYMRK acts in a signalling pathway that legume plants require to trigger the development of nodules and the uptake of fungi or bacteria into their root cells. Here we show that the induction of Actinorhiza nodulation also relies on SYMRK, consistent with the idea that both types of nodulation evolved by recruiting common signalling genes from the pre-existing AM program. We observed that *SYMRK* from different land plant lineages differs significantly in exon composition, with a “full-length” version in the nodulating clade and shorter *SYMRK* genes in plants outside this lineage. Only the most complete *SYMRK* version was fully functional in nodulation, suggesting this gene played a central role in the recruitment event associated with the evolution of intracellular root symbioses with bacteria.

## Abstract 3

Root nodule symbioses with nitrogen-fixing bacteria provide many plants with a source of nitrogen. This study uncovers evidence that changes in the gene *SYMRK* were involved in the evolution of this important biological innovation.

## Introduction

**Figure 1:**
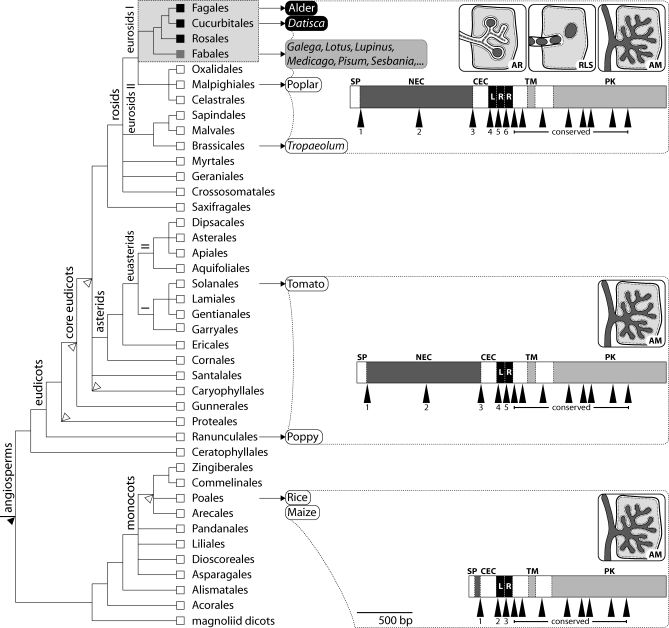
*SYMRK* Exon-Intron Structure and Root Endosymbiotic Abilities of Angiosperm Lineages All putative *SYMRK* genes encode an N-terminal signal peptide, an extracellular region with two or three LRR motifs and one imperfect LRR, a transmembrane domain, and an intracellular serine/threonine protein kinase. *SYMRK* regions encoding putative kinase domains exhibit conserved intron positions and phases. Bars illustrate the exon-intron and predicted protein domain structure of representative *SYMRK* candidates. Positions of introns are indicated by black arrowheads. Predicted protein domains are SP, signal peptide; NEC, N-terminal extracellular region; CEC, conserved extracellular region preceeding LRRs; LR(R), leucine-rich repeats; TM, transmembrane domain; and PK, protein kinase domain. Names refer to species sampled and are shaded according to their root endosymbiotic capabilities: black, endosymbiosis with *Frankia* bacteria (Actinorhiza) and AM formation; gray, endosymbiosis with rhizobia and AM formation; white, AM formation only. Pictograms symbolize AR, Actinorhiza; RLS, Rhizobium-Legume Symbiosis; AM, Arbuscular Mycorrhiza. Dashed frames have no phylogenetic implications. The cladogram depicts relationships of angiosperm orders as deduced by molecular markers [53,54]. The four orders containing nodulating taxa are shaded light gray. Squares at the tips of branches indicate the presence of taxa with particular root endosymbiotic phenotypes (colour code is as for sampled plants). Filled and white wedges indicate branches where taxa on order and family level have been omitted, respectively. Popular species designations refer to Alder, Alnus glutinosa; Poplar, *Po. trichocarpa*; Tomato, *Ly. esculentum*; Poppy, P. rhoeas; Rice, O. sativa; Maize, Z. mays.

Nitrogen limits plant growth in many terrestrial ecosystems. Evolutionary adaptations to this constraint include symbiotic associations with bacteria that are capable of converting atmospheric nitrogen into ammonium. Extracellular associations of plants with diverse groups of nitrogen-fixing bacteria are phylogenetically widespread, but only a small group evolved the ability to accommodate bacteria endosymbiotically inside cell wall boundaries. Bacterial symbionts are confined within tubular structures called infection threads, which are surrounded by a host-derived membrane that is continuous with the plasma membrane, and bound by plant cell wall–like material [1,2]. The bulk of host plants including all actinorhizal species retain the bacterial symbionts within these structures during the nitrogen-fixing stage of the symbiosis [1,3]. In the most advanced forms found exclusively among legumes (Fabales) and *Gunnera* [4], symbiotic bacteria are delimited from the host cell cytoplasm only by a plant-derived membrane in the mature stage of the symbioses. In the respective legumes, they develop into bacteroids contained in organelle-like symbiosomes, where nitrogen fixation takes place (for a recent review, see [5]). Bacterial endosymbioses in both legumes and actinorhizal plants are typically associated with the formation of novel plant organs, so-called nodules, which are root-derived in the majority of cases [6]. Nitrogen-fixing root nodule symbiosis (RNS) occurs in two major forms. Actinorhiza hosts belong to three eurosid orders (Figure 1) and nodulate with Gram-positive actinobacteria of the genus *Frankia* [7]. Legumes, on the contrary, enter specific interactions with members of a diverse group of Gram-negative bacteria, termed rhizobia. For almost a century, the extreme diversity in organ structure, infection mechanisms, and bacterial symbionts among nodulating plants obscured the fact that the nodulating clade is monophyletic, which was revealed by molecular phylogeny relatively recently [8]. The restriction of endosymbiotic root nodulation to a monophyletic group of four angiosperm orders (Figure 1) is coincident with a patchy occurrence within this clade. These observations led to the hypothesis that a genetic change acquired by a common ancestor may predispose members of this lineage to evolve nodulation endosymbiosis [8].

The molecular adaptations underlying the evolution of plant-bacterial endosymbioses are still a mystery, despite a substantial biotechnological interest in understanding the genetic differences between nodulating and non-nodulating plants. While the molecular communication between legumes and rhizobia has been studied in some detail, important clues are expected from the genetic analysis of the yet underexplored Actinorhiza.

Bacterial signalling molecules and corresponding plant receptors involved in RNS are known only for the legume–rhizobium interaction. *Frankia* signals may be biochemically distinct from rhizobial chito-oligosaccharide nodulation factors [9], which would suggest an independent mechanism of host–symbiont recognition in Actinorhiza.

Phenotypic analysis of legume mutants has revealed a genetic link between RNS and Arbuscular Mycorrhiza (AM), which is a phosphate-scavenging association between plant roots and fungi belonging to the phylum Glomeromycota [10]. AM is widespread among land plants, where forms of AM are found in representatives of all major lineages. Fossil evidence for ancient AM-like associations [11] suggests a role of this symbiosis in the colonization of land about 450 million years ago.

The link of plant–fungal and plant–bacterial endosymbioses in legumes, which involves at least seven genes [12–16] termed “common symbiosis genes” [17], inspired the idea that during the evolution of bacterial endosymbiosis, genes were recruited from the pre-existing AM genetic program [18]. However, the molecular steps involved are not clear.

## Results

To gain insight into the evolution of nitrogen-fixing root nodulation, we analysed common symbiosis genes across angiosperm lineages with different symbiotic abilities. Many, including the calcium/calmodulin kinase gene *CCaMK* [14,19], or genes encoding the predicted cation channels CASTOR and POLLUX [12,20,21], are conserved in overall domain structure. We discovered exceptional diversification among genes encoding the symbiosis receptor kinase SYMRK in different species (Figure 1). While putative SYMRK kinase domains are conserved and contain characteristic sequence motifs discriminating them from related kinases (Figure S1), the predicted extracellular portion of SYMRK occurs in at least three versions of domain composition (Figure 1 and Table 1). The longest *SYMRK* version is present in all tested eurosids, including nodulating and non-nodulating lineages. Comprising 15 exons, it encodes three leucine-rich repeat (LRR) motifs and an extended N-terminal domain of unknown function (NEC-domain, Figure 1 and Table 1). Outside of the eurosid clade, which encompasses all nodulating groups, one or more exons are absent from *SYMRK* coding sequences (Figure 1 and Table 1).

**Table 1:**
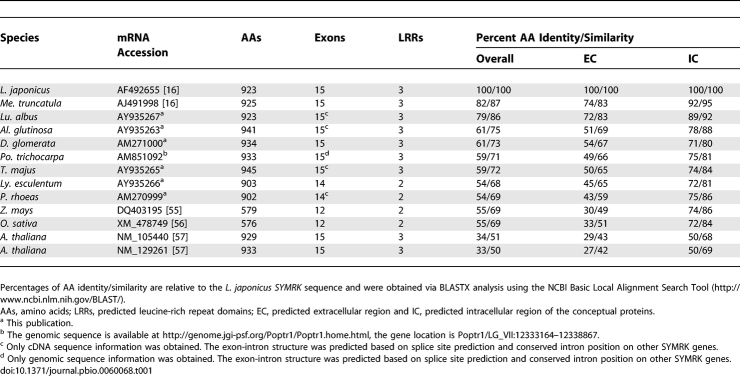
*SYMRK* Homologs, Conceptual SYMRK Proteins, and the Closest *Arabidopsis* Sequences

Genetic evidence indicates that SYMRK acts near a point of molecular convergence of AM and legume-rhizobium signalling [16,22]. The presumed ability of its diverged extracellular domain to perceive symbiosis-related signals [16] renders it a prime target for investigating the molecular adaptations underlying the evolution of RNS.

The homogenous occurrence of “full-length” *SYMRK* genes among legumes, actinorhizal plants, and non-nodulating eurosids raises the intriguing possibility that *SYMRK* is involved in the proposed genetic predisposition [8] of this clade to evolve nodulation. An important prediction following from this hypothesis is the common requirement of a full-length *SYMRK* version for all types of RNS. Furthermore, also non-nodulating members of this monophyletic clade may carry nodulation-competent versions of *SYMRK*. To test this concept, we analysed the functional capabilities of “full-length” *SYMRK* genes from symbiotically diverse eurosids.

### *SYMRK* Is Required for Actinorhiza and AM in Datisca glomerata

**Figure 2:**
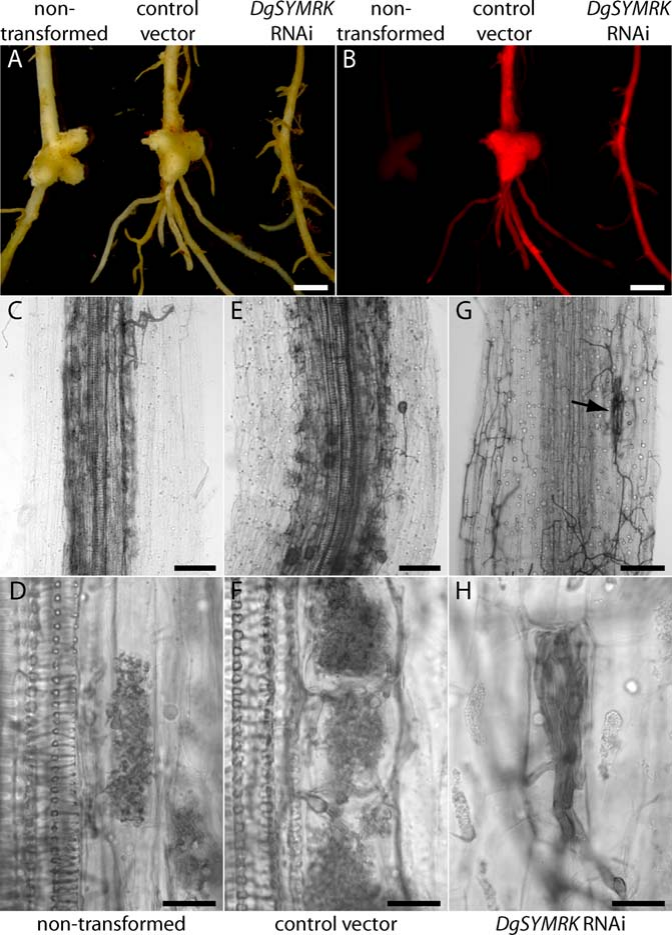
Nodulation and AM Formation Are Impaired in *DgSYMRK* Knockdown Roots Co-transformed roots express *DsRED1* as visible marker. (A and B) Nodulated wild-type root (left), control root transformed with pRedRoot lacking the silencing cassette (middle), and non-nodulated DgRNAi knockdown root (right) (A) under white light and (B) with transgenic roots showing DsRED1 fluorescence. (C–H) AM phenotype of D. glomerata (*Datisca*) wild type, transgenic control, and *DgSYMRK* knockdown roots. (C and D) Wild-type and (E and F) transgenic control roots are well colonized and show arbuscules in inner cortical cells. (G and H) Typical *DgSYMRK* knockdown root with no AM formation but extraradical mycelium and aborted fungal infections (H and arrow in G). Such features were not seen in *Datisca* wild-type or transgenic control roots and are reminiscent of those observed on *L. japonicus symrk* mutant roots (Figure 3). Roots were inoculated simultaneously with *Frankia* bacteria and *G. intraradices* (8 wk). Transgenic and regenerated nontransgenic roots of 27 (control) and 23 (*DgSYMRK* RNAi construct) plants from three independent experiments were tested. Independent transformed roots examined were *n* = 42 (control) and *n* = 55 (*DgSYMRK* RNAi). Scale bars: (A and B) 2 mm; (C, E, and G) 0.1 mm; (D, F, and H) 0.02 mm.

To investigate *SYMRK* function in Actinorhiza, we reduced root mRNA levels of D. glomerata (*Datisca*) *SYMRK* (*DgSYMRK*) via RNA-interference (RNAi)*.* Quantitative PCR following reverse transcription showed a 36%–99% reduction of *DgSYMRK* transcript levels in knockdown roots (*n* = 16) compared with vector control roots (*n* = 16). Eight weeks after inoculation with *Frankia* bacteria, no nodules were detected on *DgSYMRK* RNAi roots (Figure 2A and 2B), except for small, primordial swellings on 16% of independent transformed roots (9/55). Nonsilenced control roots of the same plants and roots transformed with a binary vector lacking the silencing cassette (transgenic control roots) showed wild type–like nodules with lobed structure typical for *Datisca* (Figure 2A and 2B). This result demonstrates that *SYMRK* is essential for Actinorhiza development in *Datisca*. In conjunction with the well-documented role of legume *SYMRK* in the interaction with rhizobia [16,23], *SYMRK* thus represents a common genetic requirement for the two types of bacterial root endosymbiosis.

**Figure 3:**
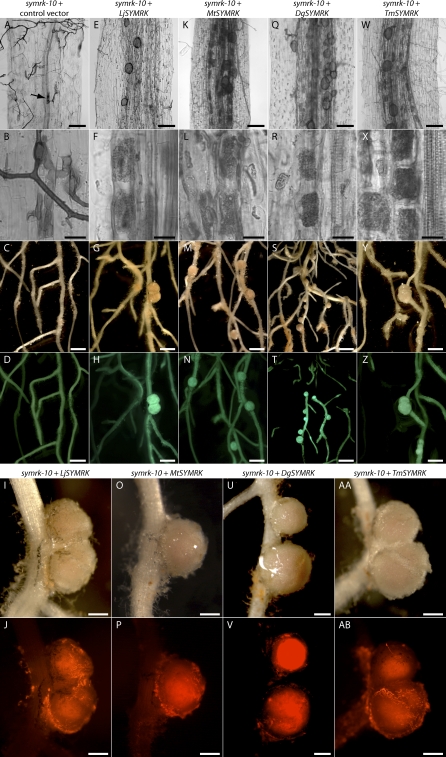
*Datisca*, *Medicago*, and *Tropaeolum SYMRK* Restore Fungal and Bacterial Endosymbioses in *Lotus symrk-10* Mutants Transgenic roots were identified via fluorescence of eGFP encoded on the transfer-DNA. (A–D) *L. japonicus* (*Lotus*) *symrk-10* roots transformed with the respective control vector lacking a *SYMRK* expression cassette. (E–AB) *Lotus symrk-10* roots transformed with *Lotus* (E–J), *Me. truncatula (Medicago)* (K–P), *D. glomerata (Datisca)* (Q–V) and *T. majus (Tropaeolum)* (W–AB) *SYMRK* under control of the *LjSYMRK* promoter region. (A, B, E, F, K, L, Q, R, W, and X) Roots co-cultivated with G. intraradices for 3 wk. (A and B) Transgenic control roots devoid of intraradical hyphae or arbuscules, with aborted fungal infection structures within epidermal cells (B and arrow in A). Roots carrying *Lotus* (E and F), *Medicago* (K and L), *Datisca* (Q and R), and *Tropaeolum* (W and X) *SYMRK* with dense fungal colonization of the inner root cortex (E, K, Q, and W) and arbuscule formation in inner cortical cells (F, L, R, and X). (C, D, G–J, M–P, S–V, and Y–AB) Root systems inoculated with M. loti expressing *DsRED* for 4 wk. (C and D) Transgenic control roots showing no nodules. *symrk-10* root systems transformed with *Lotus* (G–J), *Medicago* (M–P), *Datisca* (S–V), and *Tropaeolum* (W–AB) *SYMRK* develop nodules on transgenic roots. Nodules exhibit pink coloration in white light, indicating the presence of symbiosis-specific leghemoglobins (I, O, U, and AA) and DsRED fluorescence in inner nodule tissue indicating the presence of M. loti (J, P, V, and AB). Scale bars: (A, E, K, Q, and W) 0.1 mm; (B, F, L, R, and X) 0.02 mm; (C, D, G, H, M, N, S, T, Y, and Z) 2 mm; (I, J, O, P, U, V, AA, and AB) 0.5 mm.

To test whether *DgSYMRK* is also required for AM, we inspected *DgSYMRK* RNAi roots for AM formation with the fungus Glomus intraradices (*Glomus*)*. Datisca* wild-type roots of the same plants used for hairy root induction and independent transgenic control roots formed AM, with dense arbuscular colonization of inner cortical cells (Figure 2C–2F). In contrast, symbiotic development in *DgSYMRK* RNAi roots was strongly impaired. In 82% of independent transformed roots, no fungal infection was observed, despite the presence of extensive extraradical mycelium (Figure 2G), with those roots exhibiting strong reduction levels of *DgSYMRK* being nonsymbiotic concerning both nodulation and AM formation. Occasional infection attempts occurred but typically were aborted in the outer cell layers (Figure 2G and 2H). We conclude that similar to the situation in legumes, *SYMRK* of the actinorhizal plant *Datisca* is involved in both bacterial and fungal endosymbioses.

### *SYMRK* Does Not Mediate Specificity in Legume–Rhizobium Recognition

To determine whether *SYMRK* plays a role in the specific recognition of rhizobia by legume hosts, we tested whether L. japonicus (*Lotus*) *SYMRK* (*LjSYMRK*) can mediate nodulation in another legume, which interacts with a different rhizobial partner. The specific symbiont of *Lotus* is Mesorhizobium loti, whereas Medicago truncatula (*Medicago*) interacts with *Sinorhizobium meliloti*. *Medicago dmi2* 5P mutants exhibit a deletion in exon three of the *SYMRK* ortholog *DMI2*, leading to a frameshift and premature stop codon. *Dmi2* 5P plants form no infection threads or nodules upon inoculation with either rhizobial strain. Transgenic roots of these plants, and of wild-type control plants carrying *LjSYMRK*, formed infection threads and indeterminate, pink nodules typical for *Medicago* [24] with S. meliloti (Figure S2 and Table 2). *LjSYMRK* can therefore fully restore nodulation of *Medicago* with S. meliloti, indicating that *SYMRK* is not directly involved in determining legume–rhizobium specificity.

**Table 2:**
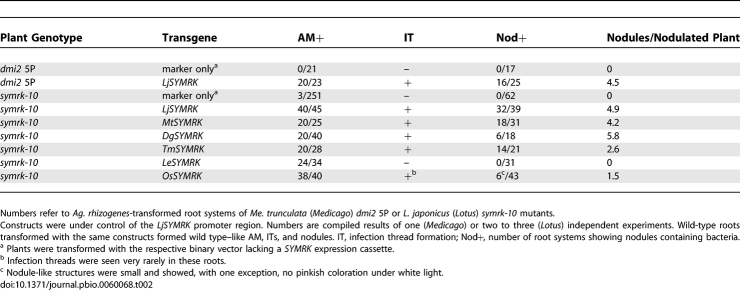
Restoration of Root Symbioses in *Medicago* *dmi2* and *Lotus symrk* Mutants Transformed with Different *SYMRK* Versions

*Medicago dmi2* 5P mutants are also impaired in AM. No arbuscules were observed within 2 wk of co-cultivation, with fungal infection being aborted at the root surface or after entry into epidermal cells (Figure S2 and Table 2). *LjSYMRK* restored the AM defect in transgenic roots of this line (Figure S2 and Table 2), demonstrating that *SYMRK* is sufficiently similar to *DMI2* to support both fungal and bacterial endosymbioses in *Medicago*.

### “Full-Length” *SYMRK* Versions from Symbiotically Distinct Eurosids Can Support Both AM and RNS in *Lotus*

To analyze the symbiotic capabilities of “full-length” eurosid *SYMRK* genes from a legume (*MtDMI2*), an actinorhizal plant (*DgSYMRK*), and the non-nodulating, AM-forming Tropaeolum majus (*Tropaeolum*; Brassicales) (*TmSYMRK*), we tested their potential to function in the *Lotus* symbiosis signalling context. We introduced these genes, under the control of the *Lotus SYMRK* promoter region, into roots of *Lotus* line SL1951–6 (*symrk-10*), which carries a *symrk* mutant allele encoding a kinase-dead SYMRK version [25,26]*.* Upon inoculation with *Glomus*, *symrk-10* roots form no AM, and fungal infections are typically associated with aberrant hyphal swellings and are aborted after entry into epidermal cells (Figure 3A and 3B, and Table 2). Interaction with M. loti is blocked at an early stage, and no infection threads or nodules form (Figure 3C and 3D, and Table 2). In *symrk-10* roots transformed with *MtDMI2*, *DgSYMRK* or *TmSYMRK* both AM and nodulation were restored, the latter involving the formation of infection threads and pink, bacteria-containing nodules (Table 2 and Figure 3) that were indistinguishable from wild-type nodules. In conclusion, consistent with a role of *SYMRK* in the predisposition to evolve RNS, we could not detect a functional diversification of the eurosid *SYMRK* version linked to features differentiating actinorhizal or legume nodulation, or to the specific recognition of bacterial symbionts. Thus, other factors, such as nod factor receptor kinases [22,27,28] or yet-unknown additional components, are likely accountable for the fine-tuning of recognition specificity in plant–bacterial endosymbioses within the eurosids.

### *SYMRK* Versions of Reduced Length Restore AM but Not RNS in *Lotus*

SYMRK from the non-nodulating eudicots Papaver rhoeas (poppy) and Lycopersicon esculentum (tomato) represent intermediate length and domain composition (Figure 1 and Table 1). To explore the symbiotic capabilities of this version, we introduced the two-LRR encoding *Le* *SYMRK* genomic sequence fused to the *LjSYMRK* promoter into *Lotus symrk-10* transgenic roots. *LeSYMRK* selectively restored AM symbiosis, whereas neither infection threads nor nodules developed upon inoculation with M. loti (Table 2 and Figure S3E–S3L).

A reduced *SYMRK* version is present in the AM-forming, non-nodulating monocots Oryza sativa (rice) and Zea maize (maize), encoding two LRRs only and a short N-terminal region, mainly covered by a single exon aligning with exon four of *LjSYMRK* (Figure 1 and Figure S1). To determine whether the short *SYMRK* version of rice, *OsSYMRK*, is sufficient for endosymbiosis formation in *Lotus*, we introduced the *OsSYMRK* genomic sequence controlled by the *LjSYMRK* promoter into *symrk-10* roots. AM formation was fully restored in these roots, whereas nodulation with M. loti was impaired (Table 2 and Figure S3M–S3Z). *OsSYMRK*-containing *symrk-10* roots inoculated with M. loti exhibited primordial swellings generally devoid of bacteria (Figure S3U–S3X). In rare cases, infection threads and small round nodules were observed, which contained bacterial colonies (Figure S3Y and S3Z). Hence, similar to *LeSYMRK*, *OsSYMRK* is compromised in supporting bacterial endosymbiosis in *Lotus*.

## Discussion

### A Molecular Link between the Two Types of RNS

In legumes, *SYMRK* is indispensable for root endosymbiosis with rhizobia and AM fungi [16,23]. We show here that this endosymbiosis gene is also required for nodulation in the actinorhizal plant *Datisca*. *SYMRK*, which is likewise essential for Actinorhiza formation of the tree species Casuarina glauca (Fagales) [29], represents the first known plant gene required for Actinorhiza, indicating a shared genetic basis of the two different types of RNS. A future task will be to determine whether further endosymbiosis genes acting in concert with *SYMRK* in legumes are also required for Actinorhiza.

### An Ancient Genetic Program for AM among Angiosperms

The ability of different *SYMRK* versions from both dicot and monocot lineages to restore AM in *Lotus* indicates a homologous nature of the AM genetic program in angiosperms. This is consistent with the observation that loss-of-function mutations in the rice version of the legume symbiosis gene *CCaMK* results in loss of AM symbiosis [30]. In *Arabidopsis*, the absence of root symbiotic capability is accompanied by a deletion of several symbiosis genes, including *SYMRK* and *CCaMK* [21,31,32].

### A Role of *SYMRK* in the Predisposition to Evolve RNS

Our survey of *SYMRK* sequences across angiosperms revealed at least three structurally distinct versions, and we could show that this polymorphism is functionally related to the root symbiotic capabilities of host plants. The variation in SYMRK domain composition is exceptional among the known common symbiosis genes. The congruence between the phylogenetic distribution of the “full-length” *SYMRK* version with the nodulating clade strongly suggests a link between *SYMRK* sequence evolution and the acquisition of endosymbiotic root nodulation with bacteria. An attractive hypothesis is that *SYMRK* sequence divergence was a critical step in mediating the recruitment of the otherwise conserved common symbiosis pathway from the pre-existing AM genetic program. Recruitment was proposed to account for the genetic link of AM and nodulation in legumes [17,18] and would make root–bacterial endosymbiosis as a whole a fascinating example for novel traits evolving on the basis of pre-existing genetic patterns.

A common feature associated with endosymbiotic bacterial infection in both actinorhizal [33] and legume hosts [34] is the formation of intracellular pre-infection threads (PITs) in host cells. These cytoplasmic structures resemble the pre-penetration apparatus (PPA) preceding fungal infection during AM formation [35]. Forming in anticipation of bacterial symbionts, PITs are thought to coordinate the uptake of bacteria and determine the spatial progression of infection through the host cell [33,34]. A similar role in guiding fungal transition through host cells in AM has been demonstrated for PPAs [35]. These developmental similarities in AM, Actinorhiza, and legume-rhizobium infection may reflect a common genetic program for endosymbiosis establishment and symbiont uptake in all three types of interactions. In AM, PPAs are not formed in mutants that are defective in certain common symbiosis genes [35]. It is therefore possible that a recruitment of AM symbiosis genes during the evolution of RNS facilitated the induction of intracellular accommodation structures in response to bacteria.

### SYMRK Domain Function and Evolution

Repetitive LRR modules have been implicated in the determination and evolution of novel recognition specificities of receptor proteins [36–38]. Interestingly, adaptive changes reflecting positive selective constraints can be traced in LRR– and NEC–encoding regions of *SYMRK* genes from different *Medicago* species, but these do not correlate with shifts in rhizobial specificity [39]. Our functional comparison of eurosid *SYMRK* versions indicates that *SYMRK* is not involved in determining recognition specificity in nodulation. However, an extended *SYMRK* version containing a set of three LRR motifs, as present in eurosid *SYMRK* genes, is required for fully supporting nodulation symbiosis of *Lotus* with M. loti. Shorter *SYMRK* versions from tomato or rice only suffice for AM. These functional differences may be caused by individual amino acid sequence polymorphisms, or alternatively, exons that are specifically required for bacterial endosymbiosis may be lacking in rice and tomato *SYMRK* versions.

At an overall structural level, exon acquisition from other genes encoding LRR or NEC-like domains [23,40] or, alternatively, retainment of exons in eurosid *SYMRK* genes, may have been an integral genetic factor in the evolution of bacterial endosymbiosis in angiosperms. The observation of small nodule-like structures on Lotus symrk mutant roots transformed with the *OsSYMRK* construct is counterintuitive, considering that the *LeSYMRK* version, which resembles the legume version more closely, does not support such developmental responses. One possible explanation may be that the nonmatching NEC region of *Le*SYMRK negatively interferes with nodulation, but not AM signalling in *Lotus*.

The NEC domain encoded by *Lotus SYMRK* exons two and three, upstream of the conserved LRR flanking region (CEC), is present across eudicot plants (Figure 1). Its function outside the nodulating group is unknown. The proposed involvement of *SYMRK* in processes such as reduction of the touch sensitivity of root hairs [41] may rely on this domain thereby imposing selective constraints. The NEC domain shows possible overall relatedness but only a low level of similarity to sequences present in the rice genome, and to sequences other than *SYMRK* candidates in genomes of dicots like *Arabidopsis* [23]. The apparent divergence observed among these potentially homologous sequences of yet unknown function is consistent with a hypothetical role as a receptor domain.

It will be a future challenge to determine the contribution of individual SYMRK LRR units as well as of the NEC domain and to resolve at the amino acid level the features of SYMRK proteins involved in conferring endosymbiotic nodulation capacity.

### Additional Components Required for Nodulation

The diversity and scattered occurrence of nodulation symbioses within the eurosid lineage suggest multiple independent origins [42]. Only a subset of the plant species carrying the “full-length” version of *SYMRK* develop root nodules, yet *SYMRK* of the non-nodulating *Tropaeolum* proved competent to support nodulation in *Lotus*. Hence, there must be additional genetic features distinguishing the nodulators. Candidate genes include those that express the legume LysM receptor kinases NFR1 and NFR5 [22,27,43], which are required for responsiveness to rhizobial lipo-chito-oligosaccharide nodulation factors, but not for AM formation. A potential relevance of LysM receptors in Actinorhiza, or the identity of alternative receptors perceiving yet unknown *Frankia* signals, remains to be determined.

## Materials and Methods

### Isolation of *SYMRK* homologues.

We used a PCR strategy employing degenerate primers to obtain *SYMRK* sequence information from diverse angiosperms, for which no genome or root-derived expressed sequence tag sequences were available. Degenerate primers for the isolation of *SYMRK* genes were positioned in regions of the coding sequence conserved among *SYMRK* candidates, but not in other similar O. sativa (rice) and A. thaliana (*Arabidopsis*) sequences. For primer sequences, see Table S1.

λ Zap cDNA libraries were available for isolation of *Ly. esculentum* (tomato) and Alnus glutinosa (alder) *SYMRK*. A cosmid clone carrying the *LeSYMRK* genomic region was isolated from a pooled tomato Cf2/9 library (kind gift of J.D.G. Jones, The Sainsbury Laboratory, United Kingdom) and shotgun sequenced.

For rapid amplification of cDNA ends (RACE) reactions, total RNA was extracted from roots of uninoculated seedlings or young plants and DNaseI treated. RT and 5′/3′RACE reactions were done using the SMART RACE kit (Clontech), following nested degenerate PCR reactions ([10 s 94 °C, 10 s 52 °C, 30 s 72 °C] × 35, 5 min 72 °C) to obtain initial sequence information.

### Construct generation for mutant complementation and *Datisca SYMRK* RNAi experiments.

For hairy root complementation assays, *SYMRK* cDNAs were amplified from root cDNA preparations (Superscript II, Invitrogen) of the respective species. Binary transformation vectors were pCAMBIA 1302 or pK7WG2D,1 [44] derivatives. L. japonicus (*Lotus*), *Me. truncatula* (*Medicago*), D. glomerata (*Datisca*), and T. majus (*Tropaeolum*) *SYMRK* coding sequences were amplified from complementary DNA using primers LjSYMRK_EC_f with LjSYMRK_PK_r, MtSYMRK_EC_f with MtSYMRK_PK_r, DgSYMRK_EC_f with DgSYMRK_PK_r and TmSYMRK_EC_f with TmSYMRK_PK_r (Table S1), respectively. An *MtSYMRK* genomic segment containing intron one was amplified from total DNA using primers MtSYMRK_EC_f with MtSYMRK_01_r (Table S1) and ligated to the 3′ fragment of the *MtSYMRK* cDNA following BstB1 digestion of both. The genomic sequences of tomato and rice *SYMRK* were amplified from total DNA using primers LeSYMRK_EC_f with LeSYMRK_PK_r and OsSYMRK_EC_f with OsSYMRK_PK_r (Table S1), respectively. A 285-bp fragment amplified with primers polyA_NOS_f and polyA_NOS_r (Table S1) from pJawohl8 RNAi (kind gift of P. Schulze-Lefert, Max Planck Institute for Plant Breeding Research, Cologne, Germany) was used as terminater in all constructs. *SYMRK* genes or coding sequences were under control of 4,970 bp of genomic sequence preceding the *LjSYMRK* translation start site. For pK7WG2D constructs, the cauliflower mosaic virus (CaMV) 35S promoter driving the Gateway-cassette was deleted Sal1(10124)-Sal1(38).

For *Datisca* RNAi experiments, a pRedRoot- [45] based binary vector encoding DsRED1 for visible selection was equipped with a pKANNIBAL [46] CaMV 35S promoter-driven hairpin construct containing 367-bp of 3′ coding and untranslated sequence of *DgSYMRK* in forward and reverse orientation, amplified with primers DgSYMRK_RNAi_f and DgSYMRK_RNAi_r (Table S1).

### Biological material and phenotyping assays.

L. japonicus wild-type ecotype B-129 Gifu and derived mutant line *symrk-10* [25] were inoculated with M. loti R7A as described [16]. Growth conditions were 24 °C constant at 16-h-light/8-h-dark cycles. For infection of *Agrobacterium rhizogenes–*induced transgenic roots in *Lotus*, M. loti MAFF 303099 expressing *DsRED* was applied at a final optical density at 600 nm (OD_600_) of 0.02 in translucent plastic boxes containing 300 ml Seramis (Mars) and 150 ml liquid Fahraeus Plant (FP) medium [47]. *Ag. rhizogenes*-transformed *Me. truncatula* A17 wild type and *dmi2* 5P (kind gift of G. Oldroyd, John Innes Centre, United Kingdom) plants were inoculated with S. meliloti 1021 carrying pBHR-mRFP [48] (OD_600_ of 0.02) in planting pots containing Seramis, and fertilized with FP medium supplemented with 50 μM KNO_3_ two times per week. For nodulation assays and simultaneous observation of infection thread (IT) formation in *Lotus* and *Medicago*, plants were harvested 4 and 5 wk post inoculation, respectively. Prior to inoculation, roots showing no eGFP fluorescence were removed. For AM phenotyping of *Lotus* and *Medicago*, plants were co-cultivated with G. intraradices BEG195 and harvested after 3 or 2 wk of co-cultivation, respectively. Root systems were stained with acidic ink as described [49]. Prior to staining of *Ag. rhizogenes–*induced root systems, roots showing no eGFP fluorescence were removed. Roots were scored AM-positive (AM+) if symbiotic structures (arbuscules and vesicles) were present, as AM-negative (AM–) if no arbuscules were present. Occasional Lotus symrk mutant roots showing vesicles not accompanied by arbuscules were scored AM–. Where complemented *Lotus symrk* mutant roots exhibited aborted infection sites in co-occurrence with successful infection and colonization events involving arbuscule and vesicle formation, roots were scored AM+. *Datisca* seeds and *Frankia* inoculum were a kind gift from K. Pawlowski (Department of Botany, Stockholm University, Sweden). *Datisca* was inoculated with compatible *Frankia* by potting in substrate with ∼1 g/l crushed nodules and with G. intraradices BEG195 by adding substrate extracted from pots of inoculated Allium schoenoprasum plants. Growth conditions were 16 h light/8 h dark at 22 °C and 60% relative humidity. Seeds of T. majus and P. rhoeas were purchased at Notcutts Garden Centres (UK). The ability to develop AM with G. intraradices was confirmed for all species involved in the study.

### *Lotus* transformation.

Transgenic roots on *Lotus symrk-10* mutants were induced using *Ag. rhizogenes* strains AR1193 [50] and LBA1334 [51] as described by Díaz et al. [52] (modified).

### *Medicago* transformation.

*Medicago* seedlings were transformed as described at http://www.isv.cnrs-gif.fr/embo01/manuels/index.html (modified), using strain *Ag. rhizogenes* AR1193 [50].

### *Datisca* transformation.

Twelve-wk-old *Datisca* plants were inoculated with *Ag. rhizogenes* strain LBA1334 [51] carrying the silencing construct by stem injection, and roots emerging at infection sites were covered with substrate. Three-wk post inoculation roots were inspected for DsRED1 fluorescence. Nonfluorescent roots were removed, and plants were repotted and grown for 8 wk. After determination of the nodulation phenotype, individual fluorescent roots were divided into two halves. One half was stained for mycorrhiza visualization, the second used for total RNA extraction (RNeasy Plant Kit, Qiagen). Quantitative RT-PCR was performed with GeneAmp5700 (Applied Biosystems) using the SuperScript III Platinum Two-Step qRT-PCR-Kit (Invitrogen). A 123-bp *DgSYMRK* fragment was amplified using primers DgqPCR_SYMRK_f with DgqPCR_SYMRK_r (Table S1). As control, polyubiquitin cDNA was amplified using primers DgqPCR_Ubi_f with DgqPCR_Ubi_r (Table S1). Representative fragments were sequenced for identity confirmation.

### Computational analysis.

Databases used for BLAST sequence search and analysis included http://www.ncbi.nlm.nih.gov/BLAST/, http://www.arabidopsis.org/Blast/, http://www.gramene.org/Multi/blastview, and http://genome.jgi-psf.org/Poptr1/Poptr1.home.html.

## Supporting Information

Figure S1 SYMRK Kinase Regions Share Several Defining Conserved Amino Acid Motifs, Which Are Absent in Similar Sequences in *Arabidopsis* and Rice Black shading indicates amino acid residues identical in all sequences, residues found in at least 50% of the sequences are shaded gray. Bars delimit predicted SYMRK protein domains. Dark blue, conserved extracellular region (CEC); black, LRRs; gray, imperfect LRR; white, juxtamembrane regions; brown, transmembrane region; green, protein kinase domain. Light blue bars with stars mark some of the regions conserved among SYMRK candidates, but not in other homologous sequences in rice and A. thaliana. Locus tags are indicated for similar sequences not regarded as SYMRK candidates. Sequences aligning with regions upstream of exon 4 of *LjSYMRK* are not included.
(96 KB DOC)

Figure S2 *LjSYMRK* Restores Nodulation and AM Formation in *Medicago dmi2* 5P Mutants Transformation assay and selection were as in Figure 3. (A, B, K, and L) *Me. truncatula* (*Medicago*) *dmi2* 5P roots transformed with the respective control vector lacking an *LjSYMRK* expression cassette.
(C–F, M, and N) *Medicago* wild-type and (G–J, O and P) *dmi2* 5P roots transformed with the *LjSYMRK* coding sequence controlled by the *LjSYMRK* promoter.
(A–J) Roots inoculated with S. meliloti expressing *DsRED* for 5 wk. (A and B) Transgenic *dmi2* 5P roots carrying the control vector, showing no nodules. (C and D) Nodules on transgenic and nontransgenic roots of a wild-type plant transformed with *LjSYMRK* and (E and F) individual nodule containing *DsRED* expressing S. meliloti bacteria. (G and H) *dmi2* 5P root system with nodule formation confined to roots transformed with *LjSYMRK*. (I and J) Nodule on an *LjSYMRK* containing *dmi2* 5P root showing bacterial DsRED expression.
(K–P) Roots co-cultivated with G. intraradices for 2 wk. (K and L) Transgenic *dmi2* 5P control roots lacking hyphal proliferation and arbuscule formation in the inner root cortex. Hyphal swellings in the root periphery (L and arrow in K) indicate abortion of fungal infections. Longer co-cultivation for 3 wk or more allowed for successful fungal infections of mutant roots, which was similarly reported for other *dmi2* mutant lines [58]. (M and N) Wild-type and (O and P) *dmi2* 5P roots transformed with *LjSYMRK* showing dense fungal colonization of the root inner cortex accompanied by arbuscule formation.
Scale bars: (A–D and G–H) 2 mm; (E–F and I–J) 0.5 mm; (K, M, and O) 0.1 mm; (L, N, and P) 0.02 mm.
(1.8 MB PDF)

Figure S3 Tomato and Rice *SYMRK* Restore AM Symbiosis in *Lotus symrk-10* Mutants, but Cannot or Only Partially Complement Bacterial Endosymbiosis Formation Transformation assay and selection were as in Figure 3. (A–D) *Lotus symrk-10* roots transformed with the respective control vector lacking a *SYMRK* expression cassette. (E–L) *Lotus* wild-type (E–H) and *symrk-10* (I–L) roots transformed with *LeSYMRK*. (M–Z) *Lotus* wild-type (M–R) and *symrk-10* (S–Z) roots transformed with *OsSYMRK*.
(A, B, E, F, I, J, M, N, S, and T) Roots co-cultivated with G. intraradices for three weeks. (A and B) Transgenic *symrk-10* control root with extraradical mycelium but no intraradical fungal colonization or arbuscule formation. Swollen hyphal structures indicative of aborted fungal infections can be observed within epidermal cells (B and arrow in A). (E and F) Wild-type and (I and J) *symrk-10* roots transformed with *LeSYMRK*, showing fungal colonization of the inner root cortex (E, I) and arbuscule formation in inner cortical cells (F, J). (M and N) Wild-type and (S and T) *symrk-10* mutant roots transformed with *OsSYMRK*, similarly showing cortical AM colonization (M, S) and arbuscule formation (N, T).
(C, D, G, H, K, L, O–R, and U–Z) Root systems inoculated with M. loti expressing *DsRED* for 4 wk. (C and D) *symrk-10* root system with transgenic control roots, showing no nodules. (G and H) and (O–R) Wild-type root systems with *M. loti–*containing pink nodules on nontransgenic and on transgenic roots carrying *LeSYMRK* or *OsSYMRK*, respectively, indicating that these transgenes do not impair nodulation in transgenic wild-type roots. (K and L) *symrk-10* root system transformed with *LeSYMRK*, showing no nodules. In a single case, one nodule primordium was observed. (U–Z) *symrk-10* root system transformed with *OsSYMRK*, showing no fully developed nodules, but nodule primordia which are mostly noncolonized by bacteria, the latter proliferating on the primordial surface (W and X). In rare cases, small nodules were observed that contained bacteria, but, with one exception, showed no pinkish coloration in white light (Y and Z).
Scale bars: (A, E, I, M, and S) 0.1 mm; (B, F, J, N, and T) 0.02 mm; (C, D, G, H, K, L, O, P, U, V) 2 mm; (Q, R, and W–Z) 0.5 mm.
(3.8 MB PDF)

Table S1 Primer Sequences (106 KB DOC)

### Accession Numbers

Sequences of *SYMRK* homologs were deposited at the EMBL Nucleotide Sequence Database (http://www.ebi.ac.uk/embl/) under accession numbers AY935263 (*Al. glutinosa*); AM271000, AM931079 (D. glomerata coding and genomic sequence, respectively); AY935267 (Lupinus albus); AY935265 (T. majus); AY935266, AY940041 (*Ly. esculentum* coding and genomic sequence, respectively); AM270999 (P. rhoeas); AM851092 (Po. trichocarpa). The GenBank (http://www.ncbi.nlm.nih.gov/Genbank/index.html) accession number for pCAMBIA 1302 is AF234298.

## Abbreviations

AM: Arbuscular Mycorrhiza
LRR: leucine-rich repeat
NEC: N-terminal extracellular region of predicted SYMRK proteins
PIT: pre-infection thread
PPA: pre-penetration apparatus
RNS: root nodule symbiosis
RACE: rapid amplification of cDNA ends
RNAi: RNA interference
