# Supplementary material for: Functional Adaptation of a Plant Receptor- Kinase Paved the Way for the Evolution of Intracellular Root Symbioses with Bacteria
Source: PLoS Biol. 2008 Mar 4;6(3):e68. doi: 10.1371/journal.pbio.0060068 (PMC2270324; doi:10.1371/journal.pbio.0060068)
Supplement: Figure S1 — Black shading indicates amino acid residues identical in all sequences, residues found in at least 50% of the sequences are shaded gray. Bars delimit predicted SYMRK protein domains. Dark blue, conserved extracellular region (CEC); black, LRRs; gray, imperfect LRR; white, juxtamembrane regions; brown, transmembrane region; green, protein kinase domain. Light blue bars with stars mark some of the regions conserved among SYMRK candidates, but not in other homologous sequences in rice and A. thaliana. Locus tags are indicated for similar sequences not regarded as SYMRK candidates. Sequences aligning with regions upstream of exon 4 of LjSYMRK are not included. (96 KB DOC) [file pbio.0060068.sg001.doc]

________________________________________________________________________________________________________________________________________________________

__________________________________________________________________________****______________________****_______________________________________________________

*L. japonicus* (359) VGVIQKMREELLLQNSGNRALESWS---GDPCILLPWKGIACDGSNGSS-VITKLDLSSSNLKGLIPSSIAEMTNLETLNISHNSFDGSVPSFPLSSLLISVDLSYNDLMGKLPESIVKLPHLKSLYFGCNEHMS-PEDPANMNSSLINTDY

*M. sativa* (362) LELIQKMREELLLHNRENEALESWS---GDPCMIFPWKGITCDDSTGSS-IITMLDLSSNNLKGAIPYFVTKMTNLQILNLSHNQFDSLFPSFPPSSLLISLDLSYNDLDGRLPESIISLPHLKSLYFGCNPYMK-DEDTTKLNSSLINTDY

*A. glutinosa* (375) VEVAWRVRNELLVSNQANAVLESWS---GDPCLPKPWQGLACALHNGSA-IITSLNLSSMNLQGSIPHSITELANIETLNMSYNQFNGSIPEFPDSSMLKSVDISHNYLAGSLPESLISLPHLQSLYFGCNPYLD-KEPQSSFN-STIHTDN

*D. glomerata* (365) VEVSLNSRDELLAYNKVNEVLKSWS---GDPCLPLPWDGLACESINGSS-VITKLDLSDHKFEGLFPFSITELPYLKTLNLSYNDFAGKVPSFPASSMLQSVDLSHNKFIGVLPESLASLPYLKTLNFGCNQFGDGNELPPNFNSSRIKTDF

*P. trichocarpa* (360) VEVIMKVRNELMLNNKENELLQSWS---GDPCFP-PWKGLKCQNISGSLPVITGLNISSSQFQGPIPASITELSYLKELNLSYNGFTGKIPEFPKSSVLTSVDLSFNDLSGSVPDSLASLTNLKTLYFGCNPLSS-TELPSNSSR--LITDS

*T. majus* (366) VNVIVNVKEELLKHNKRNVLWESWS---GDPCLPYPWDGLVCYSVNGSS-VITELNLSSRKLQGPIPSSIIQLTYLKDLNLSYNGFTGTIPSFTASSMLTSVDLRNNDLKGSLHESIGALQHLKTLDFGCNPQLD-KELPSNFKKLGLTTDK

*L. esculentum* (363) VDIMANVKKELLQQNKNNEIWKSWS---GDPCLPLPWPGLTCDRVNGTS-VITQIDLSSGGLSGPSPPSIQKLMHLRKLNISINGSSGTNS-----------------------LFTSYFTYSTRYLSSRIHISN--KLSRSIKESNITTDK

*P. rhoeas* (359) FDAITEVKDELVAQNPENELWGSWT---GDPCLPLPWEGLFCIPNNQGSLIITNLDLSWSNLQGSLPSAVTKLSNLEKLDVSHNEFVGSIP-----------------------ESFSSMPHLTRLYFGCNPQFKNDLPSSLMDRSNLTTDS

*Z. mays* (28) ADVAKRLKEVLSERNRGHEMLDSWNG-DGDPCSPSTWEGFSCEPKDGAQ-VVVKLNFSSKKLQGPIPAEIANLTELNEIHLQYNNFTGFIP-----------------------ASFSAFRHLLKLSVICNPLLN-NKQPDGFSSG-VNFSY

*O. sativa* (34) VDVGRQLREELWERNQGHEMLRSWR--DGDPCSPSPWEGFSCRWKDGNL-FVVKLNFSSKKLQGPIPAAIGNLTELDEIDLQDNNFTGSIP-----------------------ESFFDLTHLLKLSVKCNPFLN-NQLPHGLSIS-VEFSY

*Os*11g01200 (366) -ISVGSQDANIMASLVSRYPEAGWAQEGGDPCLPASWSWVQCSSEAAPR--IFSISLSGKNITGSIPVELTKLSGLVELKLDGNSFTGQIPDFTGCHDLQYIHLEDNQLTGALPPSLGELPNLKELYIQ-NNKLSGEVPQALFKKSIIFNFS

*At*1g67720 (375) --------VSVLDAIRSMSPDSDWASEGGDPCIPVLWSWVNCSSTSPPR--VTKIALSRKNLRGEIPPGINYMEALTELWLDDNELTGTLPDMSKLVNLKIMHLENNQLSGSLPPYLAHLPNLQELSIE-NNSFKGKIPSALLKGKVLFKYN

*At*2g37050 (375) --------ATVMANVASLYSSTEWAQEGGDPCSPSPWSWVQCNSDPQPR--VVAIKLSSMNLTGNIPSDLVKLTGLVELWLDGNSFTGPIPDFSRCPNLEIIHLENNRLTGKIPSSLTKLPNLKELYLQ-NNVLTGTIPSDLAKD-VISNFS

________________________________________________________________________________________________________________________________________________________

____________________________________________________________________________________________________________*******_____________________***_____________

*L. japonicus* (506) GRCK-------------GKESRF-GQVIVIGAITCGSLLITLAFGVLFVCRYRQKLIPWEGFAGKKYPMETNIIFSLPSKDDFFIKSVSIQAFTLEYIEVATERYKTLIGEGGFGSVYRGTLNDGQEVAVKVRSATSTQGTREFDNELNLLS

*M. sativa* (509) GRCK--------------GKKPKFGQVFVIGAITRGSLLITLAVGILFFCRYRHKSITLEGFGGKTYPMATNIIFSLPSKDDFFIKSVSVKPFTLEYIEQATEQYKTLIGEGGFGSVYRGTLDDGQEVAVKVRSSTSTQGTXEFDNELNLLS

*A. glutinosa* (521) GRCD-------------SNESPR-VRVSVIATVACGSFLFTVTVGVIFVCIYRKKSMPRGRFDGKGHQLTENVLIYLPSKDDISIKSITIERFTLEDIDTATENYKTLIGEGGFGSVYRGTLSDGQEVAVKVRSATSTQGTREFENELNLLS

*D. glomerata* (513) GKCD-------------HRGSPRSIQAIIIGTVTCGSFLFTVMVGIIYVCFCRQKFKPRAVFDSSRPVFMKNFIISLSSIDDHVSEPINPKDFPLEFIEDITQKYSTLIGEGGFGSVYRGTLPDGQEVAVKVRSATSTQGTREFENERKLLS

*P. trichocarpa* (505) GKCS-------------RQRSTKKTLGIVIGAITGGSFLFTLAVGMFCSCFCRNKSRTRRNFDRKSNPMTKNAVFSVASTVS---KSINIQSFPLDYLENVTHKYKTLIGEGGFGSVYRGTLPDGQEVAVKVRSSTSTQGTREFDNELTLLS

*T. majus* (513) GECG-------------SQGPKHSTRAIIISIVTCGSVLFIGAVGIVIVFFYRRRS-AQGKFKGSRHQISNNVIFSIPSTDEPFLKSISIEEFSLEYITTVTQKYKVLIGEGGFGSVYRGTLPDGQEVGVKVRSSTSTQGTREFDNELTLLS

*L. esculentum* (486) GMAN-------------VKQNSSSTHKLVIGAAVGTALLVILAIVISVVCLFKRRVMAGPKFLMRNYSITRNAVYSVPSMDTTMMKSISSRNFKLEYIEAITQNYKTLIGEGGFGSVYRGTLPDGVEVAVKVRSATSTQGIREFNNELNLLS

*P. rhoeas* (485) GKCA-------------QASKRS---LYFIGTVAGGAVFFSVAFGALFLCFYKKRR-KSRSRVEEEIQITNDVVFSIASMDSLFVKSIFIEPFSLDSIETATSKYKTMIGEGGFGSVYHGTLRNGQEVAVKVLSATSTQGTREFENELNLLS

*Z. mays* (153) GGCATQEYYSSPAEEYQSPPAVASQKVYVIGGVAGGSLACTVALGSFFVCFNKRER----RSPKKDCSSTTNPVFQECSIHN--TTNPAVQQLSLKAIQTATSNYKTMIGEGGFGAVYRGALANGQEVAVKVRSSSSTQGTREFNNELRLLS

*O. sativa* (158) GGCA-----------YHSPPGASNQRIAVIGGVAGGSLACTFALGFFFVCFDKREK----NPQKKDCSSTRNPVFEECSTHK--ATNSAVQQLSLKSIQNATCNFKTLIGEGGFGSVYRGTLAHGEEVAVKVRSTSSTQGTREFNNELRLLS

*Os*11g01200 (514) GNSDLRMG----------HSNTG-RTIVIIVCAVVGAILILVAAIVCYLFTCKRKKKS------SDETVVIAAPAKKLGSFFSEVATESAHRFALSEIEDATDKFDRRIGSGGFGIVYYGKLTDGREIAVKLLTNDSYQGIREFLNEVTLLS

*At*1g67720 (516) NNPEL------------QNEAQRKHFWQILGISIAAVAILLLLVGGSLVLLCALRKTKRADKGDSTETKKKGLVAYSAVRGGHLLDEGVAYFISLPVLEEATDNFSKKVGRGSFGSVYYGRMKDGKEVAVKITADPSSHLNRQFVTEVALLS

*At*2g37050 (515) GNLNLEK-----------SGDKG-KKLGVIIGASVGAFVLLIATIISCIVMCKSKKNNK--LGKTSELTNRPLPIQRVSSTLSEAHGDAAHCFTLYEIEEATKKFEKRIGSGGFGIVYYGKTREGKEIAVKVLANNSYQGKREFANEVTLLS

________________________________________________________________________________________________________________________________________________________

________________________*****_**_*****___***_____________________________________________________***_*_**_**_____******_______________________________**

*L. japonicus* (644) AIQHENLVPLLGYCNESDQQILVYPFMSNGSLQDRLYGEPAKRKILDWPTRLSIALGAARGLAYLHTFPGRSVIHRDIKSSNILLDHSMCAKVADFGFSKYAPQE-GDSYVSLEVRGTAGYLDPEYYKTQQLSEKSDVFSFGVVLLEIVSGR

*M. sativa* (647) AIQHENLVPLLGYCNEYDQQILVYPFMSNGSLLDRLYGEASKRKILDWPTRLSIALGAARGLAYLHTFPGRSVIHRDVKSSNILLDQSMCAKVADFGFSKYAPQE-GDSYVSLEVRGTAGYLDPEYYKTQQLSEKSDVFSFGVVLLEIVSGR

*A. glutinosa* (659) EIRHENLVPLLGHCSENDQQILVYPFMSNGSLQDRLYGEPAKRKTLDWPTRLSIALGAARGLTYLHTNAKRCIIHRDVKSSNILLDHSMCAKVADFGFSKYAPQE-GD-CVSLEVRGTAGYLDPEYYSTQQLSDKSDVYSFGVVLLEIVTGR

*D. glomerata* (652) LFRNENLVPLLGYCSENDQQILVYPFMSNGSLQDRLYGELSKRKPLDWPTRISIALGAARGLTYLHTYVGGCVIHRDVKSSNILLDQSMCAKVADFGFSKGAPQE-GD-VASLEVRGTAGYLDPEYYTSHSLSAKSDVFSFGVVLLEIICGR

*P. trichocarpa* (641) ALRHENLVPLLGYCCENDQQILVYPFMSNGSLQDRLYGEAAKRKTLDWPTRLSIALGAARGLTYLHTFSGRCIIHRDVKSSNILLDHSMNAKVTDFGFSKYAPQE-GDSGASLEVRGTAGYLDPEYYSTQHLSAKSDVFSFGVVLLEIVSGR

*T. majus* (651) TIRHENLVPLLGYCCENGQQILVYPFMSNGSLQDRLYGEAAKRKVLDWPTRLSIALGAARGLTYLHSLAGRSLIHRDVKSSNILLDQSMTAKVADFGFSKYAPQE-GDSCASLEVRGTAGYLDPEYYSTQQLSAKSDVFSFGVVLLEIISGR

*L. esculentum* (625) AITHENLVPLIGYCCENEQQILVYPFMSNSSLQDRLYGGAAKRKILDWPARLSIALGAARGLLYLHTFSERCLIHRDVKSSNILLDQSMCAKVADFGFSKYASQE-GDSGTSLEVRGTAGYLDPEYYSTQRLSAKSDVFSFGVVLLEILTGR

*P. rhoeas* (620) SIQHENLVPLLGYCCEKDQQILVYPFMSNGSLQDRLYGEAAKRKTLDWQTRLSVALGAARGLLYLHTFSGRAIIHRDVKSSNILLDHTMTAKVADFGFSKYAPQE-GDSNASLEVRGTAGYLDPEYYSTQHLSAKSDVFSFGVVLLEIITGR

*Z. mays* (299) AVWHENLVPLIGYCCEKDQQILVYPFMSNGSLQDRLYGEASKRKVLDWPTRLSVCIGAARGLVYLHNFAGRCIIHRDIKSSNILLDHSMCGKVADFGFSKYAPQE-GDSNPSMEVRGTAGYLDPEYYSTQVLSTRSDVFSFGVVLLEIVTGR

*O. sativa* (293) AVRHDNLVPLIGYCCEKDQEILVYPFMSNGSLQDRLYGEASKRKVLDWPTRLSVCIGAARGLAHLHGFAGRCIIHRDVKSSNILLDHSMCGKVADFGFSKYAPQE-GDSNASMEVRGTAGYLDPEYYSTQSLSTKSDVFSFGVVLLEIVTGR

*Os*11g01200 (649) RIHHRNLVSFLGYSQQDGKNILVYEFMHNGTLKEHLRGGPDDVKITSWVKRLEIAEDAAKGIEYLHTGCSPTIIHRDLKSSNILLDKNMRAKVADFGLSKPVVDG---SHVSSIVRGTVGYLDPEYYISQQLTEKSDMYSFGVILLELISGH

*At*1g67720 (656) RIHHRNLVPLIGYCEEADRRILVYEYMHNGSLGDHLHG-SSDYKPLDWLTRLQIAQDAAKGLEYLHTGCNPSIIHRDVKSSNILLDINMRAKVSDFGLSRQTEED--LTHVSSVAKGTVGYLDPEYYASQQLTEKSDVYSFGVVLFELLSGK

*At*2g37050 (653) RIHHRNLVQFLGYCQEEGKNMLVYEFMHNGTLKEHLYGVVPRDRRISWIKRLEIAEDAARGIEYLHTGCVPAIIHRDLKTSNILLDKHMRAKVSDFGLSKFAVDGT--SHVSSIVRGTVGYLDPEYYISQQLTEKSDVYSFGVILLELMSGQ

______________________________________________________________________________________________________________________________________________________

**_____**_*********____________________________________________________***___****_*****_______________________________________________________________

*L. japonicus* (795) EPLNIKRPRTEWS-LVEWATPYIRGSKVDEIVDPGIKGG-YHAEAMWRVVEVALQCLEPFSTYRPSMVAIVRELEDALIIENNASEYMKSIDSLG----------GSNRYSIVIEKR------VLPSTTSTAE---STITTQSLSHPQPR

*M. sativa* (798) EPLNIKRPRIEWS-LVEWAKPYIRASKVDEIVDPGIKGG-YHAEALWRVVEVALQCLEPYSTYRPCMVDIVRELEDALIIENNASEYMKSIDSLG----------GSNRYSIVMDKR---------ALPSTTSTAESTITTQTLTHPQPR

*A. glutinosa* (809) EPLNIHRPRNEWS-LVEWAKAYIRDSQIDEMVDPSIRGG-YHAEAMWRVVEVASTCIESDAASRPLMIDILRELDEALIIETNASEYMRSIDSLGT---------SSNRFSIVMEKR------IVLPPISSSPSEPSPILPQGPSPPQPR

*D. glomerata* (802) EPINVRMPRSEWS-LVEWAKPYIRQSRIDEIVDPSIKGG-YHAEAMWRVVEVAVACIEPFSAYRPCMADIVRELEDALIIENNASEYMRSIDSMYSLG-------GSNRFSVGNDKK-------MGLPPTPCPTEPS-PITHDLAPPEPR

*P. trichocarpa* (792) EPLNIHRPRNEWS-LVEWAKPYIRESRIDEIVDPGIKGG-YHAEAMWRVVEVALVCIEPFSAYRPCMTDIVRELEDALIIENNASEYMKSIDSLGGYSLGGSNRFGSNRFSISTDKK------IALSPPVPTPPDPSPINTQALAPLEPR

*T. majus* (802) EPLNIHRPRNEWS-LVEWAKPYIRESRIDEIVDPTIKGG-YHAEAMWRVVEVALACIEPFSAHRPCMADIVRELEDGLIIENNASEYMKSIDSIGGYSFGG----GSNRFSIIVTDKEKKTIPPPPSPPTPNPSEPSPVNTQALTPPEPR

*L. esculentum* (767) EPLNINKPRNEWS-LVEWAKPLIRSSRVEEIVDPTIKGG-YHGEALWRVVEVALACTETYSTYRPCMADIVRELEDALIIENNASEYLKSLDSFG----------GSNRFSVERSIV---------LPPIKSQTEPS-SLLSKPAPPQPR

*P. rhoeas* (771) EPLNIHRPRSEWS-LVEWAKPLVQESRIEDLVDPSIKAG-YNAEAMWRVVEVAITCLEPFSAYRPCMSVIARELEDALIIEINASEYMKSIDSFG----------GSHRWSFADKKI------VLPAPTTPSTTEPSPIISQALAPPEPR

*Z. mays* (450) EPLDVKRPRHEWS-LVEWAKPYIREYKIEEMVDPGIKGQ-YCSEAMWRVLEVASVCTEPFSTFRPTMEDVLRELEDALIIENNASEYMRSIESTGTL--------GSNRYLSIDRKM-------FASGSARIE---SMKGNLQAMPSLPR

*O. sativa* (444) EPLDVQRPRDEWS-LVEWAKPYIREYRIEEIVDPGIKGQ-YCSEAMWRVLEVASACTEPFSTFRPSMEDVVRELEDALIIENNASEYMRSIESTGTL--------GSNRYLSIDRKM-------FASGSARFASFDATKGHLQTMPSLPG

*Os*11g01200 (798) EPISNDNFGLHCRNIVEWARSHMESGDIHGIIDQSLDAG-YDLQSVWKIAEVATMCVKPKGVLRPSISEVLKEIQDAIAIELQRELPSSIHHL---------------MSKTSPSEAV----NTTGSVQDLEQ---NASFDELLMRPGLR

*At*1g67720 (805) KPVSAEDFGPELN-IVHWARSLIRKGDVCGIIDPCIASN-VKIESVWRVAEVANQCVEQRGHNRPRMQEVIVAIQDAIRIERGNENGLKSSSS-------------SSSKAQSSRK----------TLLTSFLELESPDISRNSLAPAAR

*At*2g37050 (803) EAISNESFGVNCRNIVQWAKMHIDNGDIRGIIDPALAEDDYSLQSMWKIAEKALLCVKPHGNMRPSMSEVQKDIQDAIRIEKEALAARGGISD---------------EFSRSSAHSS----SLNMGMLDLAGSQSYVSIDESVLQPTAR
