# Supplementary figures and images for: Functional Adaptation of a Plant Receptor- Kinase Paved the Way for the Evolution of Intracellular Root Symbioses with Bacteria
Source: PLoS Biol. 2008 Mar 4;6(3):e68. doi: 10.1371/journal.pbio.0060068 (PMC2270324; doi:10.1371/journal.pbio.0060068)

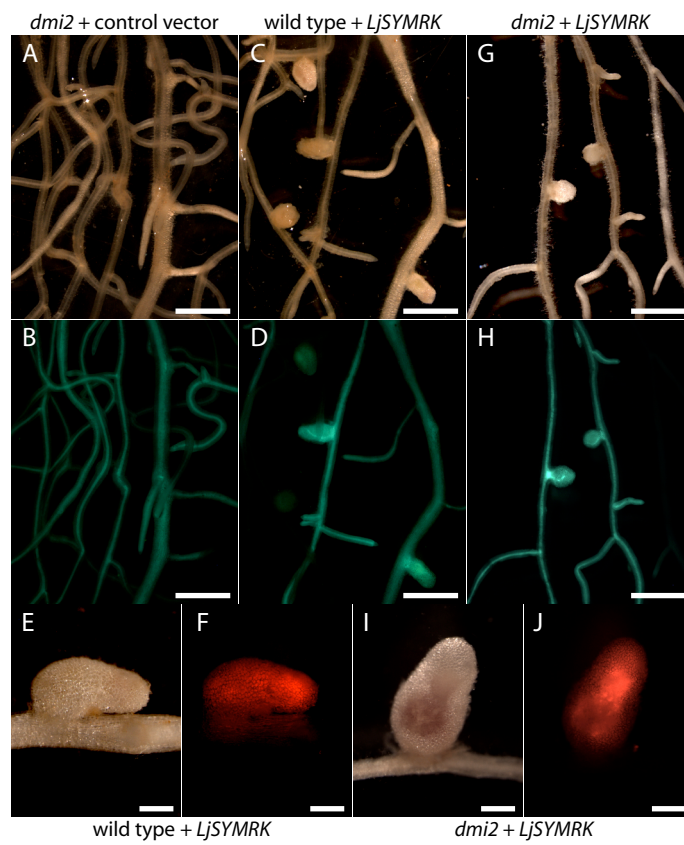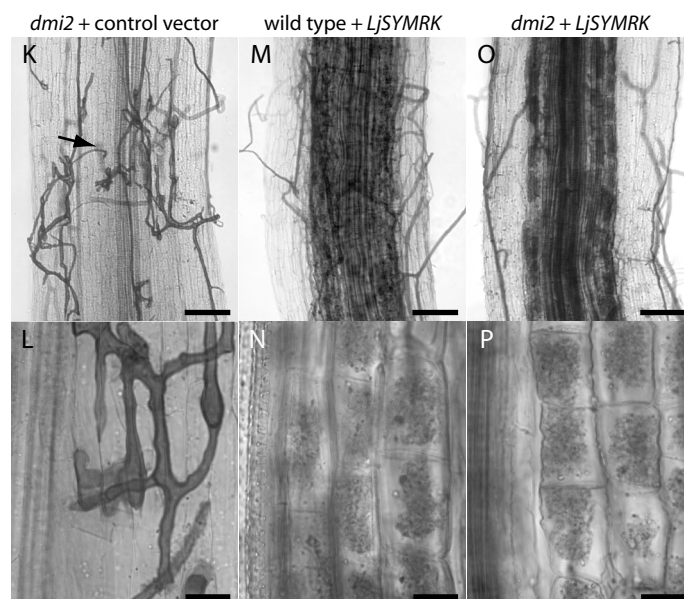

Supplement: Figure S2 — Transformation assay and selection were as in Figure 3. (A, B, K, and L) Me. truncatula (Medicago) dmi2 5P roots transformed with the respective control vector lacking an LjSYMRK expression cassette. (C–F, M, and N) Medicago wild-type and (G–J, O and P) dmi2 5P roots transformed with the LjSYMRK coding sequence controlled by the LjSYMRK promoter. (A–J) Roots inoculated with S. meliloti expressing DsRED for 5 wk. (A and B) Transgenic dmi2 5P roots carrying the control vector, showing no nodules. (C and D) Nodules on transgenic and nontransgenic roots of a wild-type plant transformed with LjSYMRK and (E and F) individual nodule containing DsRED expressing S. meliloti bacteria. (G and H) dmi2 5P root system with nodule formation confined to roots transformed with LjSYMRK. (I and J) Nodule on an LjSYMRK containing dmi2 5P root showing bacterial DsRED expression. (K–P) Roots co-cultivated with G. intraradices for 2 wk. (K and L) Transgenic dmi2 5P control roots lacking hyphal proliferation and arbuscule formation in the inner root cortex. Hyphal swellings in the root periphery (L and arrow in K) indicate abortion of fungal infections. Longer co-cultivation for 3 wk or more allowed for successful fungal infections of mutant roots, which was similarly reported for other dmi2 mutant lines [58]. (M and N) Wild-type and (O and P) dmi2 5P roots transformed with LjSYMRK showing dense fungal colonization of the root inner cortex accompanied by arbuscule formation. Scale bars: (A–D and G–H) 2 mm; (E–F and I–J) 0.5 mm; (K, M, and O) 0.1 mm; (L, N, and P) 0.02 mm. (1.8 MB PDF) [file pbio.0060068.sg002.pdf]

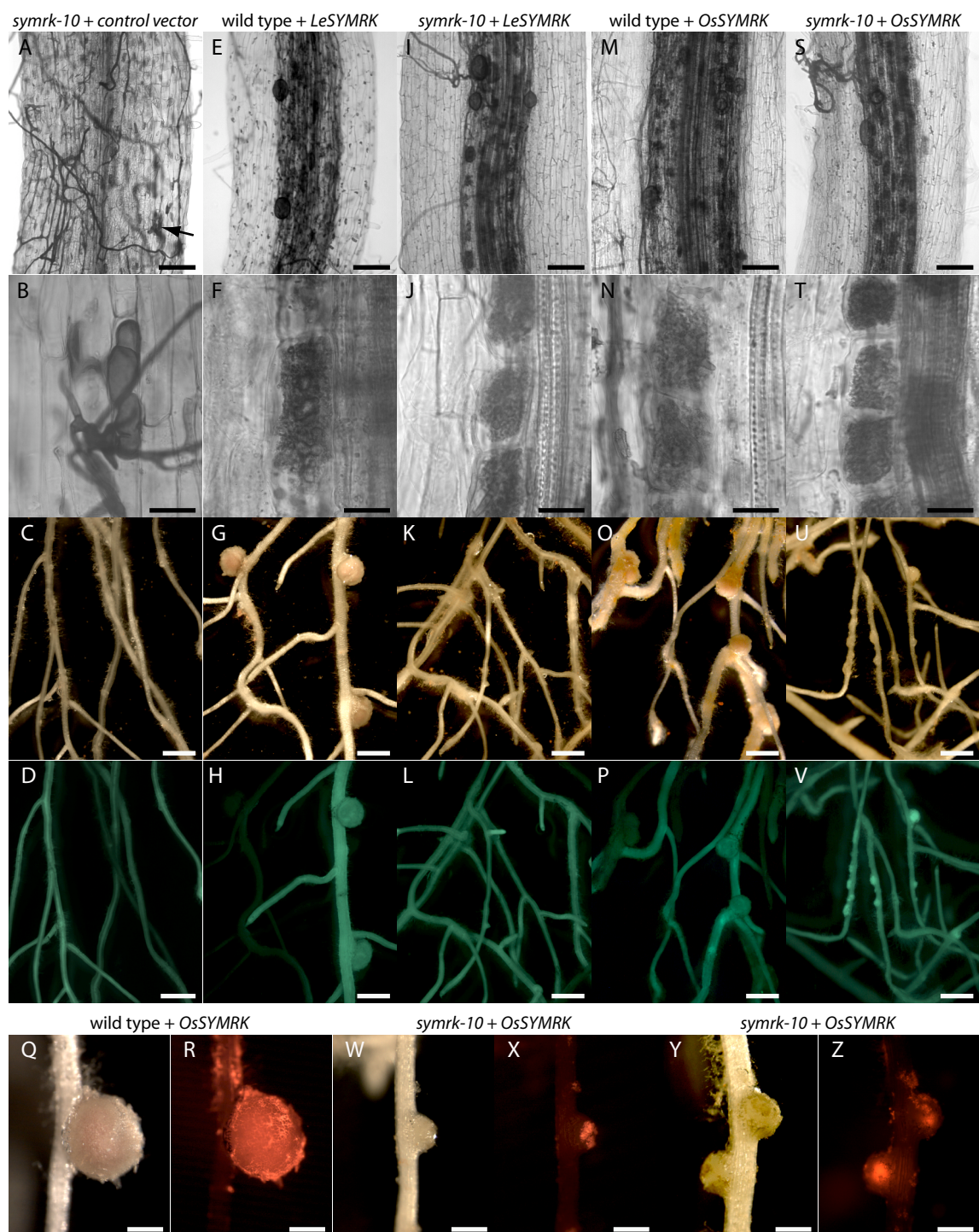

Supplement: Figure S3 — Transformation assay and selection were as in Figure 3. (A–D) Lotus symrk-10 roots transformed with the respective control vector lacking a SYMRK expression cassette. (E–L) Lotus wild-type (E–H) and symrk-10 (I–L) roots transformed with LeSYMRK. (M–Z) Lotus wild-type (M–R) and symrk-10 (S–Z) roots transformed with OsSYMRK. (A, B, E, F, I, J, M, N, S, and T) Roots co-cultivated with G. intraradices for three weeks. (A and B) Transgenic symrk-10 control root with extraradical mycelium but no intraradical fungal colonization or arbuscule formation. Swollen hyphal structures indicative of aborted fungal infections can be observed within epidermal cells (B and arrow in A). (E and F) Wild-type and (I and J) symrk-10 roots transformed with LeSYMRK, showing fungal colonization of the inner root cortex (E, I) and arbuscule formation in inner cortical cells (F, J). (M and N) Wild-type and (S and T) symrk-10 mutant roots transformed with OsSYMRK, similarly showing cortical AM colonization (M, S) and arbuscule formation (N, T). (C, D, G, H, K, L, O–R, and U–Z) Root systems inoculated with M. loti expressing DsRED for 4 wk. (C and D) symrk-10 root system with transgenic control roots, showing no nodules. (G and H) and (O–R) Wild-type root systems with M. loti–containing pink nodules on nontransgenic and on transgenic roots carrying LeSYMRK or OsSYMRK, respectively, indicating that these transgenes do not impair nodulation in transgenic wild-type roots. (K and L) symrk-10 root system transformed with LeSYMRK, showing no nodules. In a single case, one nodule primordium was observed. (U–Z) symrk-10 root system transformed with OsSYMRK, showing no fully developed nodules, but nodule primordia which are mostly noncolonized by bacteria, the latter proliferating on the primordial surface (W and X). In rare cases, small nodules were observed that contained bacteria, but, with one exception, showed no pinkish coloration in white light (Y and Z). Scale bars: (A, E, I, M, and S) 0.1 mm; (B [file pbio.0060068.sg003.pdf]
