# Supplementary material for: Functional Adaptation of a Plant Receptor- Kinase Paved the Way for the Evolution of Intracellular Root Symbioses with Bacteria
Source: PLoS Biol. 2008 Mar 4;6(3):e68. doi: 10.1371/journal.pbio.0060068 (PMC2270324; doi:10.1371/journal.pbio.0060068)
Supplement: Table S1 — (106 KB DOC) [file pbio.0060068.st001.doc]

**Supplementary Table**

Table S1. Primer Sequences

|  | **Primer Name** | **Sequence 5´ to 3´** |
| --- | --- | --- |
| A | ALIEN_r | gaatccatagatctcatatattcagaagcrttrttytc |
|  | EAM_r | aaagcaacttcaacaactctccacadngcytc |
|  | EWA_f | ccaagacatgaatggtctctggtngartgggc |
|  | GREP_f | ttctgctggaaattgttactggamgngarccnyt |
| B | Dg3RACE_f | ggcagtggcgtgtatcgaaccc |
|  | Dg5RACEinn_r | ggactcacaagccaggccatcccagg |
|  | Dg5RACEout_r | gttgggaggaagctcattgccatcgcc |
|  | Le3RACE_f | tgcatggcgggacattgtaagaga |
|  | Le5RACEinn_r | ccatgataccctcccttgatagt |
|  | Le5RACEinn_r | cactctccacagcgcctca |
|  | Pr3RACE_f | agaagcaatgtggagggtggtt |
|  | Pr5RACEinn_r | ccacattgcttctgcattataccctgctt |
|  | Pr5RACEout_r | caacttcaaccaccctccacattgctt |
|  | Tm3RACE_f | catgcatggctgacattgttcgtg |
|  | Tm5RACEinn_r | gtaacccccttttatggtcggat |
|  | Tm5RACEout_r | gctacttctaccactcgccacatt |
| C | LjSYMRK_EC_f | TTATTTATCGATGatggagttaccagc |
|  | LjSYMRK_EC_r | ctctcatcttctgaatcactcccactatgctctcaaacccttcagttgc |
|  | LjSYMRK_exon4_f | gcaactgaagggtttgagagcatagtgggagtgattcagaagatgagag |
|  | LjSYMRK_PK_r | ATTAAACCTAGGTAATAAGTCGACctatctcggctgtgggtgag |
|  | MtSYMRK_EC_f | TTATTTATCGatgatggagttacaagttattaggatatttag |
|  | MtSYMRK_01_r | cagaaatctatgtagtctttggtggc |
|  | MtSYMRK_PK_r | ATTAAACCTAGGTAATAAGTCGACctatctcggttgagggtgtgac |
|  | DgSYMRK_EC_f | TTATTTATCGatgatgatggaaggattgcataattg |
|  | DgSYMRK_PK_r | ATTAAACCTAGGTAATAAGTCGACtcatctgggttcaggaggagccaag |
|  | TmSYMRK_EC_f | TTATTTATCGatgatgatggaaagactcgac |
|  | TmSYMRK_PK_r | ATTAAACCTAGGTAATAAGTCGACctatcttggttcaggaggagtc |
|  | LeSYMRK_EC_f | TTATTTATCGatggaagtagataattgctggaac |
|  | LeSYMRK_PK_r | ATTAAACCTAGGTAATAAGTCGACcagcatttaccttggttgtggag |
|  | OsSYMRK_EC_f | TTATTTATCGatggccgcccgcttcg |
|  | OsSYMRK_PK_r | AAATAACCTAGGctaccccggaagcgaaggca |
|  | polyA_NOS_f | AATAAACCTAGGatcagcttgcatgccggtcg |
|  | polyA_NOS_r | AAATAAGTCGACctagagtcaagcagatcgttcaaac |
| D | DgSYMRK_RNAi_f | GGATCGATGGTACCatgcagaggcaatgtgga |
|  | DgSYMRK_RNAi_r | GCTCTAGACTCGAGtcactctttcataatttcccaaaaggt |
| E | DgqPCR_Ubi_f | atgcagatytttgtgaagac |
|  | DgqPCR_Ubi_r | accaccacgragacggag |
|  | DgqPCR_SYMRK_f | tgtgtgctaaggtggcagactttgg |
|  | DgqPCR_SYMRK_r | aactatggctcgtgtagtactcgggatc |

(A) Degenerate oligonucleotide primers used for identification of *SYMRK* candidates. (B) Nondegenerate primers used in 3´ and 5´ RACE reactions to isolate full-length cDNA sequences of *SYMRK* homologs. (C) Nondegenerate primers used to generate *SYMRK* constructs for hairy root complementation experiments. Upper case characters in the sequences indicate restriction enzyme sites or artificial sequence framing these. (D) Nondegenerate primers used for the preparation of the *DgSYMRK* RNAi construct. (E) Degenerate and nondegenerate primers used for expression level analysis of the indicated genes via qPCR in *Datisca*. Primer orientation is indicated by _r, complementary and _f, forward.
